# The Liver Can Deliver: Utility of Hepatic Function Tests as Predictors of Outcome in COVID-19, Influenza and RSV Infections

**DOI:** 10.3390/jcm12093335

**Published:** 2023-05-08

**Authors:** Einat Ritter, Eden Shusterman, Lior Prozan, Orli Kehat, Ahuva Weiss Meilik, Oren Shibolet, Jacob Nadav Ablin

**Affiliations:** 1 Department of Gastroenterology and Liver Diseases, Tel Aviv Medical Center, 6 Weizmann Street, Tel Aviv 64239, Israel; 2 Department of Internal Medicine H, Tel Aviv Medical Center, 6 Weizmann St., Tel Aviv 64239, Israel; 3 I-Medata AI Center, Tel Aviv Medical Center, 6 Weizmann St., Tel Aviv 64239, Israel

**Keywords:** COVID-19, liver function tests, cholestatic, hepatocellular, prognosis

## Abstract

Background: liver test abnormalities have been described in patients with Coronavirus-2019 (COVID-19), and hepatic involvement may correlate with disease severity. With the relaxing of COVID-19 restrictions, seasonal respiratory viruses now circulate alongside SARS-CoV-2. Aims: we aimed to compare patterns of abnormal liver function tests in patients suffering from COVID-19 infection and seasonal respiratory viruses: respiratory syncytial virus (RSV) and influenza (A and B). Methods: a retrospective cohort study was performed including 4140 patients admitted to a tertiary medical center between 2010–2020. Liver test abnormalities were classified as hepatocellular, cholestatic or mixed type. Clinical outcomes were defined as 30-day mortality and mechanical ventilation. Results: liver function abnormalities were mild to moderate in most patients, and mainly cholestatic. Hepatocellular injury was far less frequent but had a strong association with adverse clinical outcome in RSV, COVID-19 and influenza (odds ratio 5.29 (CI 1.2–22), 3.45 (CI 1.7–7), 3.1 (CI 1.7–6), respectively) COVID-19 and influenza patients whose liver functions did not improve or alternatively worsened after 48 h had a significantly higher risk of death or ventilation. Conclusion: liver function test abnormalities are frequent among patients with COVID-19 and seasonal respiratory viruses, and are associated with poor clinical outcome. The late liver tests’ peak had a twofold risk for adverse outcome. Though cholestatic injury was more common, hepatocellular injury had the greatest prognostic significance 48 h after admission. Our study may provide a viral specific auxiliary prognostic tool for clinicians facing patients with a respiratory virus.

## 1. Introduction

Abnormal liver function tests are reported in up to half of patients with COVID-19 infections, mainly among those with severe disease, older age and pre-existing liver injury [1]. The pattern of liver injury is frequently hepatocellular and includes a rise in alanine aminotransferase (ALT) or aspartate aminotransferase (AST), rather than an elevation in bilirubin, gamma-glutamyl transpeptidase (GGT) or alkaline phosphatase (ALP) [2].

Several mechanisms underlying liver injury have been proposed, and the pathogenesis is probably multifactorial. SARS-CoV uses the angiotensin-2 converting enzyme (ACE2) receptor to enter host cells. ACE2 is expressed in the hepatobiliary system, in both hepatocytes and, more abundantly, on the cholangiocytes [3,4]. Moreover, ACE II receptors are upregulated in cirrhotic livers [5]. Direct cellular damage may pertain to the cholestatic pattern of hepatic injury, seen in some but not in the majority of COVID-19 patients [6]. In an autopsy analysis from COVID-19 patients, numerous non-specific findings were observed, such as microvesicular steatosis and mild inflammation in the lobular and portal area, but direct viral presence was not detected [7].

The fulminant inflammatory response characteristic of COVID-19, which includes cytokine dysregulation and immune cell activation, has been suggested to propagate liver injury [8]. Moreover, there is an association between poor outcome, hepatic injury and IL-6 levels [9]. Additional factors may contribute to liver damage, including micro-thrombotic injury, hemodynamic changes and drug-induced liver injury.

Aside from the specific hepatitis viruses (HAV, HBV, HCV, etc.) other viruses have direct cytopathic effects on the liver, such as EBV and CMV. Hepatic injury has also been described in association with respiratory viruses. Thus, for example, SARS-CoV-2, which caused the previous coronavirus epidemic, was found in >41% of liver tissue samples [1,10]. On the other hand, liver involvement during influenza and RSV infection has been poorly studied and is generally considered to be uncommon. Though acute hepatitis and liver failure has been described in children with influenza A [11], it is usually presumed that influenza is not hepatotropic, and liver chemistry abnormalities are usually related to immune response, hypotension and multiorgan involvement. Papic et al. compared hepatic abnormalities in records of patients diagnosed with H1N1 influenza during the 2009 pandemic with seasonal influenza. They found that liver function abnormalities were more common among pandemic 2009 H1N1 than during seasonal influenza A. Hepatic involvement correlated with hypoxemia and higher CRP levels, though no characteristics of hypoxic liver damage were noted. Liver abnormalities were usually mild and self-limiting [12].

Recently, Shafran et al. compared liver function tests in SARS-CoV2 to influenza. This work reviewed 1737 hospitalized patient’s records. They observed mild to moderate elevation in liver tests, mainly transaminases, with an earlier peak among influenza patients. For both viruses, hepatic injury correlated with worse clinical outcomes, a finding which was suggested to be the result of acute infection per se, rather than being unique to a specific virus [13].

We aimed to compare the pattern of liver injury in COVID-19 with two other common respiratory viruses: respiratory syncytial virus (RSV) and influenza virus (A and B), as well as to determine its effect on various clinical outcomes.

## 2. Methods

### 2.1. Study Design

We conducted a retrospective single center study at the Tel Aviv Sourasky Medical Center. We searched in the microbiology laboratory database for positive reverse transcription polymerase chain reaction (RT-PCR) nasal swabs for RSV and influenza (A, B) between 2010–2020, and for SARS-CoV-2 during 2020. Clinical, demographic and laboratory findings were obtained from patient’s medical charts. The study was approved by the Institutional Review Board of the Tel Aviv Sourasky Medical Center (TLVMC).

### 2.2. Patients

Patients included in the study were adults (>18 years old) who had positive RT-PCR for RSV or influenza (A or B) or SARS-CoV-2, admitted at TLVMC between 1 January 2010 to 1 December 2020. Those with co-infection of any two viruses were excluded.

### 2.3. Data Collection

Data were extracted from patient’s medical records. Demographic and baseline patient’s information included age, sex and Charlson comorbidity index (CCI). A total of 3.5% of patients had underlying liver disease according to CCI, and were excluded. Vital signs on admission included blood pressure, heart rate, body temperature and O_2_ saturation. Laboratory test results included complete blood count, routine chemistry and liver function test, coagulation as well as C-reactive protein (CRP) and ferritin. The primary outcomes measured were poor clinical outcome, which is a composite score consisting of mortality after 30 days, need for intubation and mechanical ventilation.

### 2.4. Microbiology

The diagnosis of the different viral infections was undertaken by PCR from nasopharyngeal and pharyngeal swabs. RNA extraction was done using the easyMAG^®^ system (BioMérieux, Marcy Étoile, France). The diagnosis of SARS-CoV-2 was done by RT-PCR using mostly the Allplex™ 2019-nCoV Assay (Seegene). The diagnosis of Influenza A and B and RSV was done using the Simplexa™ Flu A/B & RSV kit (DiaSorin) or the Seeplex^®^ RV7 kit (Seegene).

### 2.5. Liver Test Parameters and Abnormalities

We extracted liver chemistry from medical charts at the patients’ admission, as well as the maximal value during hospitalization.

Liver test abnormalities were defined as elevation of the following liver tests: ALT > 35 U/L, AST > 40 U/L, GGT > 28 U/L, ALP > 116 U/L and total bilirubin (TBIL) > 1.2 mg/dL. We then classified liver abnormalities as hepatocellular, cholestatic and mixed type. Patients who had elevated AST and/or ALT above ×3 the upper limit of normal (ULN) were classified as hepatocellular type. Those whose GGT and/or ALP were raised above twice the ULN were classified as cholestatic type, and mixed type referred to a combination of both ALT/AST elevation above 3× ULN and GGT/ALP above 2× ULN.

### 2.6. Statistical Analyses

Patients’ characteristics were summarized as counts and percentages for categorical variables, and median and interquartile range for continuous variables. Pearson’s chi-square tests and Kruskal–Wallis tests were used to compare the three respiratory infections. To evaluate the relationship between liver injuries or temporal dynamics of liver enzymes and clinical outcomes, multivariate logistic regression models were applied. Statistical calculations were performed using the SPSS 25.0 software (SPSS Inc., Chicago, IL, USA).

## 3. Results

A total of 4140 patients were included in our analysis, of which 1624 were confirmed with COVID-19 infection, 2056 with influenza (A and B) and 460 with RSV infection.

Baseline and demographic characteristics including sex, age, vital signs at admission and Charlson comorbidity score were compared among the three groups (Table 1).

Patients with RSV were significantly older (median 79 years, *p* < 0.001) and had a higher comorbidity score (median 5.0, *p* < 0.001). Oxygen saturation on admission was similar among the three groups, while mean body temperature was significantly higher in the COVID-19 group (mean 37.4 °C, *p* < 0.001). COVID-19 patients suffered the highest rate of mortality compared with RSV and influenza (19%, 14% and 9%, respectively) as well as higher rates of mechanical ventilation (11%, 8% and 7%, respectively).

### 3.1. Liver Function Abnormalities

Median and maximal values of liver enzymes and liver function tests are summarized in Table 1. COVID-19 patients presented with higher levels of cholestatic injury compared with RSV and Influenza (24%, 19% and 17%, respectively). Furthermore, overall rates (throughout hospitalization) of cholestatic, mixed or hepatocellular injury were higher in the COVID-19 group (29% vs. 23% vs. 20% for cholestatic injury, 11% vs. 8% vs. 6% for mixed injury and 3% vs. 2% and 2% for hepatocellular injury in COVID-19, RSV and influenza, respectively).

### 3.2. Correlation with Clinical Outcome

Table 2 summarizes the prognostic value of hepatocellular, mixed and cholestatic injury at admission. After adjustment for age and sex, and comorbidities, cholestatic injury, which was the most common in our cohort, was associated with poor clinical outcome in COVID-19 (odds ratio 2.05, *p* < 0.01) and influenzas (odds ratio 1.7, *p* = 0.002), but not RSV. Hepatocellular injury had the highest odds ratio for adverse outcome in RSV, COVID-19 and influenza (odds ratio 5.29, 3.45 and 3.1, respectively, *p* < 0.05).

### 3.3. Temporal Pattern of Liver Enzymes and Prognostic Value

**Figure 1:**
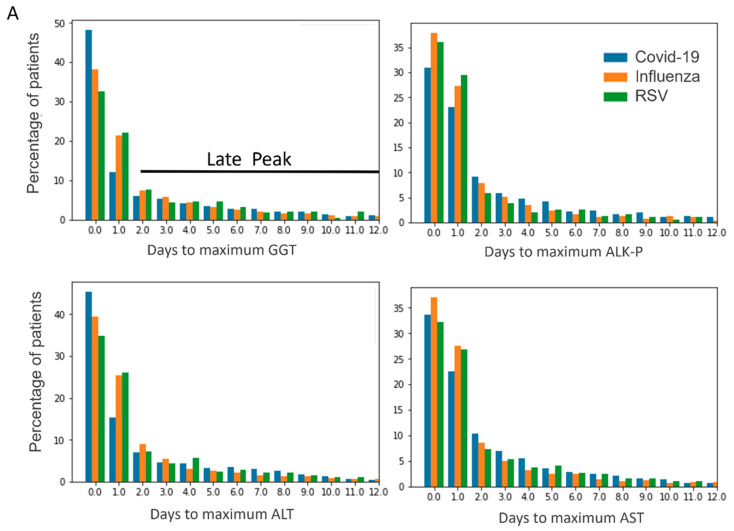

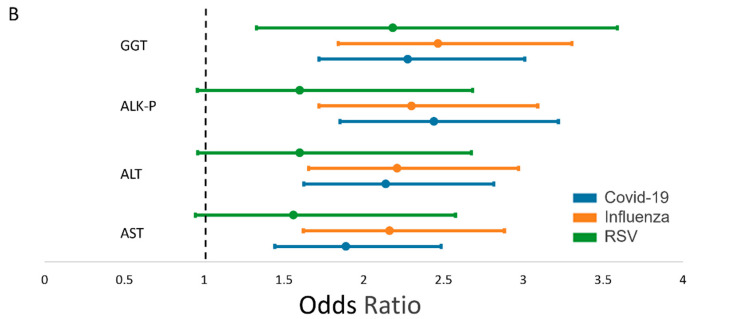
(**A**) Temporal pattern of liver enzymes in COVID-19, influenza and RSV. Patients reaching maximum values after 48 h were considered as late peakers (black line) (**B**). Predictive value of late peak of hepatocellular and cholestatic enzymes across the different viruses. The figure depicts adjusted odds ratio (point) and confidence intervals (95%) of adverse outcome for early versus late peakers, Wald Chi-square test (COVID-19 blue; influenza orange; RSV green).

The temporal pattern for the rise in AST and ALT function tests has been shown to differ between COVID-19 and influenza [13], with an earlier rise in ALT and AST for influenza patients. Here, we present the temporal pattern for the rise of both cholestatic and hepatocellular enzymes (GGT, ALKP, AST, ALT, Figure 1). Across all enzymes, the vast majority of patients reached max levels within the second day of hospitalization. A similar temporal pattern was observed among all three viruses, with highest rates of maximal values in the first day of hospitalization. Observing this pattern, two groups of patients emerge: early and late peakers. The first group (early peakers) reached their maximal liver enzyme level within 48 h, whereas the second group (late peakers) consisted of patients whose enzyme levels remained high, continued to or started to rise after 48 h. We examined the difference in adverse outcome between the two groups. Using multivariate logistic regression, we compared the risk for adverse outcome between the groups, after adjustment for sex, age and comorbidities. In order to address the confounding impact of the length of hospitalization on the results (where patients in a worse state will have longer hospitalization and hence more and later liver tests) the regression model also included the length of stay as an independent variable. In COVID-19 and influenza infections, late peakers in both hepatocellular and cholestatic patterns had an increased risk for adverse outcome of mortality and need for mechanical ventilation (for example, COVID-19 patients with a late rise in ALT have an OR 2.1 (CI 1.6–2.8) for adverse outcome). RSV patients with a persistent high level or a retarded increase in GGT have a twofold risk for adverse outcome (OR 2.1 CI (1.3–3.6)). A similar trend was evident for the other liver enzymes in RSV, though they did not reach statistical significance.

## 4. Discussion

Since the outbreak of the COVID-19 pandemic, social distancing and personal protection measures (facial masks, etc.) have dramatically lowered the rates of infection with other seasonal respiratory viruses [14]. Nevertheless, with the spread of vaccinations these measures are less common and seasonal viruses will return to circulate alongside SARS-CoV-2 [15], posing a diagnostic and therapeutic challenge. By the end of 2021, there was a clear increase in patients diagnosed with the influenza virus in Israel as well as in the USA and Europe, most with H3N2/A [16].

In the present work, we used a large cohort of patients diagnosed with influenza (n = 2100), RSV (n = 460) and COVID-19 (n = 1600) in order to describe the pattern of liver injury in these viruses and its prognostic value at admission and during hospitalization.

Cholestatic injury was the most common liver injury at presentation and in general, with higher levels in COVID-19 (24%) compared to RSV and influenza. When reviewing reports for liver injury in COVID-19, abnormal liver function was documented in 4–53% of patients [14,15], predominantly hepatocellular, and usually mild to moderate (up to five times ULN) [17,18]. The variability in liver function abnormalities may result from different definitions. Though common, liver impairment is usually not a prominent feature of COVID-19 illness, and can be described as a “bystander “ during a systemic viral infection or sepsis [1]. In RSV, hepatitis has been described in pediatric patients [19] and especially in those needing ventilation [20]. Direct evidence of viral liver infection has been reported as well [21], yet liver injury is believed to be multifactorial [21]. However, these are all very small-scale studies and, to our knowledge, our data is the first to be based on a large population of adults. Regarding influenza, a large retrospective cohort of hospitalized patients was analyzed and compared to a cohort of COVID-19 patients. The reported occurrence of acute hepatitis or liver failure was 0.5% in influenza compared to 1.6% in COVID-19, which is similar to rates of liver injury for COVID-19 reported in other large cohorts [22]. Several reports, including animal models of influenza and human patients, have generally failed to cultivate influenza virus from damaged hepatic tissue [23]. An enhanced immune response, on the other hand, has been shown to correlate with liver injury in influenza [12,24], including viral specific CD-8 expansion in the liver. Based on the “sterile” nature of liver injury, it is considered to represent collateral damage caused by an aberrant immune response in severe cases of influenza and other non-hepatotropic viruses [25,26].

In the current study we compared three different respiratory viruses. The evident similar pattern of injury across the three viruses may shed light on the more prominent underlying mechanism of liver injury. The presence of ACE2 receptors on cholangiocytes has led to the hypothesis that the liver injury observed in COVID-19 may be due to viral invasion into hepatic and mainly cholestatic cells [27,28,29]. The higher frequency of cholestatic injury in our study may support this notion. On the other hand, the similar pattern across viruses (which do not have the same hepatotropism) may suggest that a systemic inflammatory response accounts for the observed injury [30,31].

One possible explanation for the different outcome of patients with and without hepatic injury may be attributed to the effect of such injury on the use of COVID-19 specific medications, i.e., the need to avoid specific medications in the presence of liver injury. This may be the case for medications such as tocilizumab and remdesivir which are relatively contraindicated in the presence of severe hepatic impairment. Other medications such as dexamethasone and Oseltamivir, however, are not implicated as hepatotoxic and thus cannot explain the divergent outcomes between patients with and without hepatic injury on presentation [32,33,34,35].

The prognostic value of liver injury has been described for COVID-19, and recently for influenza as well [36,37]. In the current study, we have analyzed the prognostic value of liver injury at a well-defined and clinically relevant time point—upon presentation—within 24 h. Although cholestatic injury was the most common injury, it had a lower prognostic value then hepatocellular or mixed liver injury (Table 1). This difference held true for COVID-19 and influenza but did not reach statistical significance for RSV. This disparity was most evident in the RSV cohort in which, while cholestatic injury at admission did not bear a statistically significant risk for adverse outcome, patients presenting with a hepatocellular injury had a fivefold risk for adverse outcome. Therefore, at admission, liver injury in general and hepatocellular specifically are ominous markers, which should prompt clinical scrutiny.

As evident in the earlier work of Shafran et al. for COVID-19 and influenza, as well as and in our work for all three viruses, the vast majority of patients reach their maximal liver function tests within 48 h. In influenza and COVID-19, patients whose liver tests did not decrease after 48 h had an increased risk for adverse outcome, the most significant being ALKP for COVID-19 (OR-2.4) and GGT for influenza (OR 2.4). For RSV, a continuous rise or plateau in GGT levels was associated with a twofold risk for adverse outcome. Notably, all patients, even with normal levels of liver enzymes, entered this analysis. Thus, we were able to utilize the temporal pattern of the liver function tests in order to define a second prognostic “checkpoint” 48 h after hospitalization.

The current study included a large number of patients with clinical and laboratory data, between 2010–2020 at a tertiary medical center.

## 5. Limitations

Several limitations should be noted, the first of which concerns the retrospective observational nature of the study.

In addition, since there were very few cases of influenza in Israel during the COVID-19 pandemic and during the study period, it may reflect different periods of time. Additionally, during the first few months of the COVID-19 pandemic, patients with mild symptoms were referred to the E.R., which may cause selection bias. Indeed, as seen in Table 1, the cohort of COVID-19 patients is healthier with fewer comorbidities, and thus a liver injury in these patients may represent a more severe disease. Additionally, the lockdowns and social distancing measures undertaken at the start of the COVID-19 pandemic led to a decrease in other infections and exposures, which could lead to liver injury for other reasons [38].

Thirdly, we had no previous medical records, nor did we have information regarding baseline liver enzymes, current or previous medical treatment or underlying liver disease. Moreover, we did not have documentation of treatment during the hospitalization, which may lead to liver dysfunction. We partially overcame this by excluding patients with cirrhosis or chronic hepatitis according to the Charleson comorbidity index. Finally, it should be noted that the data presented in the current study include COVID-19 patients hospitalized during 2020, and the applicability of these results to more recent variants of the SARS-CoV-2, such as Omicron, is uncertain.

## 6. Conclusions

In the current study, we present laboratory-based incidences of liver injury of a large retrospective cohort of respiratory viruses (SARS-CoV-2, influenza and RSV). The results demonstrate that, while cholestatic injury is common at presentation, hepatocellular injury carries the most ominous prognostic significance in this setup. We describe different prognostic values of different liver functions at a second clinically relevant review point, 48 h after admission (a time by which results of nasal swabs are available). This study’s findings could have a significant impact on patient care. In a large cohort study, we were able to define different prognostic values of liver functions at a specific time point, which carries negative prognosis.

This information can serve as a tool for clinicians in identifying and monitoring patients at risk. Finally, the relatively similar pattern of systemic liver injury in these common respiratory viruses sheds light on the emerging notion of viral sepsis [39].

## Figures and Tables

**Table 1:** Baseline demographic features, clinical and laboratory findings *.

	COVID-19 (n = 1624)	Influenza (n = 2056)	RSV (n = 460)	
Age (years) *	69 (55–81)	74 (58–83)	79 (69–86)	*p* < 0.001
Charlson Comorbidity Index *	4 (2–6)	4 (2–6)	5 (4–7)	*p* < 0.001
**Vital Signs**				
O2 Saturation (%) *	96 (93–98)	97 (93–99)	96 (93–99)	*p* < 0.005
Heart Rate at Admission (beats/min) *	87 (77–99)	92 (80–106)	88 (77–103)	*p* < 0.001
Body Temperature (°C) *	37.4 (36.8–38)	37.1 (36.7–37.8)	37.1 (36.7–37.8)	*p* < 0.001
Systolic BP (mmHg) *	135 (120–150)	135 (117–152)	140 (123–160)	*p* < 0.01
Diastolic BP (mmHg) *	75 (66–84)	74 (64–83)	74 (65–87)	*p* < 0.05
CRP (mg/L) *	69.9 (20.34–142.96)	48.79 (21.11–113.7)	51 (20.05–112.36)	*p* < 0.001
**Baseline Liver Enzymes**				
ALT (U/L) *	24 (15–39)	22 (15–33)	20 (13–29)	*p* < 0.001
AST (U/L) *	32 (23–49)	30 (22–45)	26 (19–35)	*p* < 0.001
ALK (U/L) *	70 (55–91)	69 (55–90)	75 (60–98)	*p* < 0.001
GGT (U/L) *	33 (20–66)	27 (16–53)	29 (18–60)	*p* < 0.001
Bilirubin (mg/dL) *	0.41 (0.26–0.62)	0.33 (0.2–0.51)	0.36 (0.23–0.55)	*p* < 0.001
**Maximal Liver Enzymes**				
ALT (U/L) *	32 (20–62)	26 (18–44)	24.5 (16–40)	*p* < 0.001
AST (U/L) *	40 (26–69)	34 (24–54)	30 (22–44)	*p* < 0.001
ALK (U/L) *	79 (61–108)	74 (58–102)	83 (64–109)	*p* < 0.001
GGT (U/L) *	44 (24–99)	34 (19–70)	42 (21–88)	*p* < 0.001
Bilirubin (mg/dL) *	0.60 (0.42–0.85)	0.48 (0.3–0.7)	0.48 (0.31–0.71)	*p* < 0.001
Hepatocellular Injury (%) ^X^	3.08	2.48	1.74	*p* = 0.24
Cholestatic Injury (%) ^X^	29.13	19.75	23.48	*p* < 0.001
Mix Injury (%) ^X^	11.45	6.42	8.26	*p* < 0.001
**Outcomes**				
Death (%)	0.19	0.09	0.14	*p* < 0.001
Mechanical Ventilation (%)	0.11	0.07	0.08	*p* < 0.001
Length of Hospital Stay (days) *	5 (2–10)	4 (2–9)	5 (3–12)	*p* < 0.001

* Continuous Variables Are Presented as Medians and Inter-Quartile Range. ^X^ Definitions of abnormal liver testing: hepatocellular injury—AST and/or ALT > 3× ULN; cholestatic injury—ALKP and/or GGT > 2× ULN; mixed injury—combination of both hepatocellular and cholestatic injury. COVID-19, coronavirus disease 2019; RSV, respiratory syncytial virus; BP, blood pressure; CRP, C-reactive protein; AST, aspartate transaminase; ALT, alanine transaminase; ALK, alkaline phosphatase; GGT, gamma-glutamyltransferase; ULN, upper limit of normal.

**Table 2:** Association of abnormal liver test result at admissions with poor clinical outcome.

	COVID-19		Influenza		RSV	
	OR	*p* Value	OR	*p* Value	OR	*p* Value
Mixed	2.83	<0.001	3.3	<0.001	3.33	0.019
Cholestatic	2.05	<0.001	1.7	0.002	1.57	0.13
Hepatocellular	3.45	<0.001	3.1	<0.001	5.29	0.023

Logistic regression with age, gender and Charlson comorbidity Index. OR, odds ratio; RSV, respiratory syncytial virus.

## Data Availability

The database generated and analyzed during the current study are available from the corresponding author on reasonable request.
